# Belief Bias Effect in Older Adults: Roles of Working Memory and Need for Cognition

**DOI:** 10.3389/fpsyg.2019.02940

**Published:** 2020-01-23

**Authors:** Daoqun Ding, Yang Chen, Ji Lai, Xiyou Chen, Meng Han, Xiangyi Zhang

**Affiliations:** ^1^Department of Psychology, School of Education Science, Hunan Normal University, Changsha, China; ^2^Cognition and Human Behavior Key Laboratory of Hunan Province, Hunan Normal University, Changsha, China; ^3^Changsha Experimental Middle School, Changsha, China

**Keywords:** belief bias, syllogistic reasoning, older adults, need for cognition, working memory

## Abstract

Belief bias is the tendency in syllogistic reasoning to rely on prior beliefs rather than to fully obey logical principles. Few studies have investigated the age effect on belief bias. Although several studies have recently begun to explore this topic, little is known about the psychological mechanisms underlying such an effect. Accordingly, we investigated belief bias in older and young adults and explored the roles of working memory (WM) and need for cognition (NFC) in the relationship between age and reasoning performance. We found that older adults showed a lower accuracy rate compared with young adults when conclusion believability and logical validity were incongruent. However, older adults showed a higher accuracy rate compared with young adults when conclusion believability and logical validity were congruent. The results indicated that in comparison with young adults, prior beliefs hampered logical reasoning more significantly in older adults under incongruent conditions and boosted logical reasoning more significantly under congruent conditions. Moreover, the logic index in older adults was significantly lower than in young adults, and the interaction index of believability and validity in older adults was significantly below zero. Furthermore, NFC mediated the age effect on reasoning performance under the two conditions. By contrast, WM mediated the age effect on reasoning performance only under incongruent conditions and did not act as a mediator under congruent conditions.

## Introduction

### Belief Bias Effect

Belief refers to an understanding about the contents of reasoning based on prior knowledge and experience. In syllogistic reasoning, people do not fully follow the principles of logic, and the reasoning process is often biased by beliefs (Evans et al., 1983, 2001). When conclusion believability does not conflict with beliefs, people are likely to endorse this argument, and syllogistic reasoning is boosted by beliefs. However, when conclusion believability conflicts with beliefs, people are less likely to endorse the argument, and beliefs will hamper syllogistic reasoning (Dube et al., 2010; Trippas et al., 2013, 2018).

Researchers generally used syllogistic reasoning tasks in investigating belief bias by manipulating conclusion believability and logic validity (Dube et al., 2010; Klauer and Kellen, 2011; Trippas et al., 2013). Gilhooly et al. (1993) reported that participants showed a higher accuracy when the syllogistic tasks were presented visually than when they were presented verbally, and syllogistic performance was interfered by concurrent random-number generation but not by concurrent articulatory suppression or concurrent tapping. They argued that the central executive component of working memory (WM) plays an important role in syllogistic tasks. Copeland and Radvansky (2004) showed that WM capacity is positively correlated with syllogistic reasoning, and the larger capacity one keeps, the better reasoning performance one will have. Thus, they argued that a larger WM capacity is helpful for syllogistic reasoning. Trippas et al. (2013) showed that participants with high cognitive ability performed more accurately with unbelievable conclusions than believable conclusions when time was unlimited. However, participants with low cognitive ability performed no differently between the two conditions and showed only a response bias to believable conclusions. Thus, discrimination was suggested to be different for individuals with different cognitive abilities, and the reasoning performance of participants with low ability could be explained partially by response bias (Trippas et al., 2013). Therefore, focusing on the influence of individual difference was worth pursuing when investigating belief bias.

### Age Effect on Belief Bias

Although belief bias has been established empirically, only three studies have been conducted regarding the age effect on belief bias to date. De Neys and Van Gelder (2009) found that the accuracy rate of reasoning for children is lower than for young adults and that the accuracy rate of reasoning for young adults is higher than that of older adults when beliefs and logic are incongruent (incongruent conditions). This finding indicates that beliefs affect syllogistic reasoning more significantly in children than young adults and more significantly in older adults compared with young adults. However, reasoning performance is unaffected when beliefs and logic are congruent (congruent conditions) (De Neys and Van Gelder, 2009). The previous researchers proposed the hypothesis of belief inhibition to explain these results. Specifically, people must inhibit the influence of beliefs in incongruent conditions, and the inhibitory ability of young adults is better those that of children and older adults; moreover, young adults show a higher accuracy rate than children and older adults. However, inhibition is not assumed to play a role under congruent conditions, and age does not influence reasoning performance. Gilinsky and Judd (1994) showed that the magnitude of the age effect on reasoning performance is only partially diminished by controlling WM and vocabulary. Moreover, the age effect on reasoning is significantly associated with bias produced by a conflict between beliefs and logic. Tsujii et al. (2010) used near-infrared spectroscopy to investigate the inferior frontal cortex (IFC) activity related to belief bias in older and young adults. Accordingly, the activation in the right IFC was shown to be stronger than that in the left IFC in young adults. However, a hemispheric difference did not exist in older adults. Another finding was that reasoning accuracy has a significant positive correlation with IFC activation in both hemispheres for older adults, whereas correlation is significant only in the right hemisphere for young adults. These findings indicate that the right IFC is important in accomplishing incongruent reasoning in young adults; however, older adults will recruit the left IFC to compensate for the age-related decline in the inhibition process (Tsujii et al., 2010). In sum, although some researchers have begun exploring age differences in belief bias, little is known about the psychological mechanism.

### WM and Belief Bias

Working memory has an important effect on the reasoning process (Kyllonen and Christal, 1990). Tsujii and Watanabe (2010) speculated that the decrease in WM among older adults might contribute to the stronger belief bias in older adults compared with in young adults. De Neys and Van Gelder (2009) assumed that high WM reflects a good belief inhibition process. Several studies have suggested that WM capacity is a strong index for predicting reasoning performance (Ackerman et al., 2005; Krumm et al., 2009). Robison and Unsworth (2017) proposed that WM capacity might affect the degree of belief bias in individual difference. Therefore, the present study explores the role of cognitive ability (i.e., WM capacity) in the relationship between age and syllogistic reasoning performance.

### Need for Cognition (NFC) and Belief Bias

Several researchers have argued that the influence of cognitive ability and the effect of cognitive motivation should be considered when investigating reasoning and judgment processes (Hess et al., 2012; Samanez-Larkin and Knutson, 2015). NFC refers to the intrinsic motivation to process information, which has a crucial influence on the reasoning process (Cacioppo et al., 1996; Bruinsma and Crutzen, 2018). Individuals with high NFC tend to engage strongly in cognitive processes requiring thinking positively (Nair and Ramnarayan, 2000) and seek additional information when faced with problems such as judging and reasoning (Furnham and Thorne, 2013). By contrast, individuals with low NFC tend to rely on prior knowledge and dislike thinking on their own (Dickhäuser et al., 2009). Stupple et al. (2011) divided participants into three response groups on the basis of their propensity to endorse logically normative conclusions. They found that the low-logic group showed rapid responses, the medium-logic group showed slower responses, and the high-logic group showed relatively unbiased responses that came at the cost of increased reasoning times, especially with invalid-believable conclusions. Furthermore, age-related change in NFC occurred over time, and older adults had lower NFC levels compared with young adults (Bruinsma and Crutzen, 2018). Therefore, the effect of NFC in the relationship between age and syllogistic reasoning performance is explored in the present study.

### Present Research

Although several studies have investigated belief bias, knowledge of how age influences belief bias remains limited. Recently, although a few researchers have begun investigating the age effect on belief bias, little is known about the psychological mechanism underlying such an effect. Hence, the present study aims to investigate the belief bias in older and young adults and whether WM and NFC mediate the relationship between age and belief bias. On the basis of dual-process theories, human reasoning is affected by two systems, namely, heuristic and analytic systems. The former is mainly driven by prior knowledge and beliefs, and it is assumed to operate unconsciously and immediately trigger a response. The latter allows reasoning according to logical standards, and it is believed to operate consciously and to be controlled and relatively slow (Evans, 2003; De Neys, 2006; Bago and De Neys, 2017). Some studies have revealed that individuals with high WM are more likely to use analytic strategies and calculate responses correctly (Gilhooly et al., 1993; Copeland and Radvansky, 2004). Markovits et al. (2002) showed that WM is associated with analytic reasoning because it allows individuals to develop models of arguments and thus manipulate them to answer correctly. Furthermore, WM resources are known to decline with age (Gilinsky and Judd, 1994; Salthouse, 2003). Hence, older adults should be less likely to use analytic strategies and be easily affected by prior beliefs, whereas young adults should be more likely to use analytic strategies and be less affected by prior beliefs (Klaczynski et al., 1997; Stanovich and West, 2000; De Neys, 2006; Novak and Mather, 2007). Older and young adults might have different performances in syllogistic reasoning. Therefore, we expect that older adults will show a stronger belief bias compared with young adults under incongruent conditions. We hypothesize that older adults will show a lower accuracy rate compared with young adults under incongruent conditions. We will also examine whether an age effect exists on syllogistic reasoning under congruent conditions.

On the basis of selective processing theories (for a review, see Evans, 2007), belief bias is driven by the operation of heuristic and analytic processes. The former process is assumed to accept believable and reject unbelievable conclusions. However, the latter process is assumed to be biased by the believability of conclusions, and reasoners are regarded as operating in a “satisficing” manner. Hence, for a believable conclusion, a satisficing search is launched for a single mental model supporting the conclusion, whereas, for an unbelievable conclusion, a satisficing search is launched for a single mental model refuting the conclusion. Although a search for a counterexample model is motivated by unbelievable contents when conclusions are valid, such a model cannot be found, and the effect of belief bias is restricted. However, models that support and refute such conclusions exist when conclusions are invalid, which results in a high level of fallacious acceptance of invalid-believable problems and a high level of correct refusal of invalid-unbelievable problems (Stupple et al., 2011). Furthermore, cognitive abilities and motivations are believed to decline with age (Gilinsky and Judd, 1994; Bruinsma and Crutzen, 2018). Therefore, we hypothesize that older adults will show a lower accuracy rate relative to young adults in invalid-believable problems. We also examine whether an age effect exists in reasoning performance in other problem types.

## Materials and Methods

### Participants

A total of 45 older adults (19 females, age range 60–78 years, mean ± SD = 63.67 ± 4.05 years) and 48 young adults (32 females, age range 18–25 years, mean ± SD = 21.71 ± 2.37 years) participated in this study. According to the law on the protection of the rights and interests of the Chinese elderly, citizens who are over 60 years older are called “elders” in China. All the elderly participants were high school graduates or above to ensure that they could fully understand the experimental task, and each participant had to correctly answer all practice trials before they began the formal experiment. Written informed consent was obtained from all participants involved in the study. This study was approved by the Ethics Committee of Hunan Normal University. Each participant received a fee of 30 yuan for their participation. All participants had normal intelligence, and they were in good health without clinical histories of physical or mental illness.

### Design

A 2 (age group: older adults or young adults) × 2 (reasoning type: congruent or incongruent) mixed design was conducted in this study, where age group was a between-participants factor, whereas reasoning type was a within-participants factor.

### Materials

#### Syllogistic Reasoning Task

We adopted a modified version of the syllogistic reasoning task of De Neys and Van Gelder (2009). On the basis of the concept of subordination, we designed 24 experimental materials of categorical syllogism, which were divided into two types according to whether the logical validity and the conclusion believability were congruent (12 congruent [6 valid–believable, 6 invalid–unbelievable] and 12 incongruent trials [6 valid–unbelievable, 6 invalid–believable]). In each trial, the participants could see three sentences. The first two sentences were the premises, and the third sentence was the conclusion; the premises and the conclusion were separated by a line. Participants were required to infer whether the conclusion was correct according to the logic of syllogism. Table 1 presents an example of the syllogisms. The conclusion believability was rated on a five-point scale (1 = very unbelievable, 5 = very believable) based on a pilot study conducted among 47 additional young participants (29 females, age range 18–25 years, mean ± SD = 21.74 ± 1.55 years) and 41 older participants (15 females, age range 60–83 years, mean ± SD = 69.17 ± 6.24 years). The results indicated that no differences existed in the believability ratings for each item between the older and young participants, *p*_*s*_ ≥ 0.093 (see Supplementary Materials for more details). The middle item of each syllogism was replaced by the English letter “A” to control premise believability (Trippas et al., 2013). The participants were definitely informed that the English letter “A” in the middle term of each syllogism referred to a nonsensical term.

**TABLE 1:** Reasoning types used in the experiment.

	Conclusion	
Syllogism	Believable	Unbelievable
Valid	Some birds are A *No A are sparrows* Therefore, some birds are not sparrows	No birds are A *Some A are sparrows* Therefore, some birds are not sparrows
Invalid	Some sparrows are A *No A are birds* Therefore, some sparrows are not birds	No sparrows are A *Some A are birds* Therefore, some sparrows are not birds

#### NFC Scale

The 18-item NFC scale, which was developed by Cacioppo et al. (1984), is widely used for measuring cognitive motivation (Cacioppo et al., 1996; Nair and Ramnarayan, 2000; Fortier and Burkell, 2014; Bruinsma and Crutzen, 2018). In the present study, a Chinese version of the 18-item NFC scale introduced by Kuang et al. (2005) was used to assess NFC. Each item was coded from 1 (strongly disagree) to 7 (strongly agree). An example of an item used in the questionnaire was, “I would prefer complex to simple problems.” The internal consistency was 0.89, the split reliability was 0.90, and the test–retest reliability was 0.86 in the Chinese version of this scale. The Cronbach’s coefficient alpha value was 0.96 in the present study.

#### Operation Span (OSPAN) Task

**FIGURE 1:**
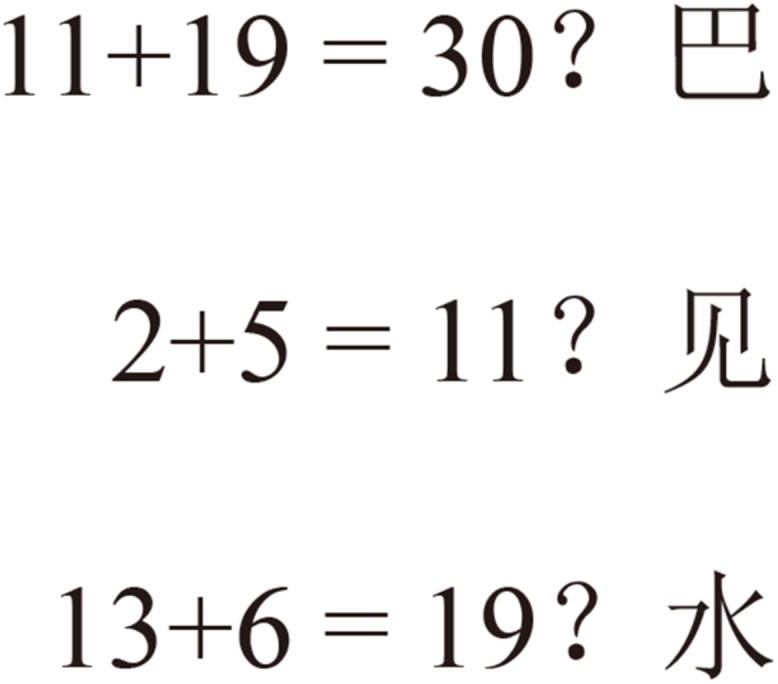
Illustration of the OSPAN task. An example of a set of three operation-word strings. The different sets of operation-word strings in length were presented in a pseudo-random order. The participants were instructed to press the “F” key with their left index finger if they thought the equation was correct and the “J” key with their right index finger if they thought otherwise.

The OSPAN task developed by La Pointe and Engle (1990) was used to assess WM capacity. In the OSPAN task, the participants were asked to solve a series of mathematical operations interleaved with irrelevant words to memorize. Each set of operation-word strings consisted of three, four, or five items (see Figure 1), and the participants observed one item at a time. The participants read the equations as quickly as possible, indicated whether the given result was correct, and then immediately read the word. After their responses, the next item was presented. The sequence continued until question marks appeared, which served as a reminder for the participants to recall all the words presented in this set. The participants entered the words on the computer in the order in which they were displayed. The OSPAN score was the sum of the recalled words in the correct order. Four sets of each length (from three to five operation-word pairs) were shown, and the scores ranged from 0 to 48. The data from the participants would have been excluded if the correct rate of equation judgment was less than 87.5% (Kane and Engle, 2003). In the present study, no data from participants were excluded from the analyses.

### Procedure

**FIGURE 2:**
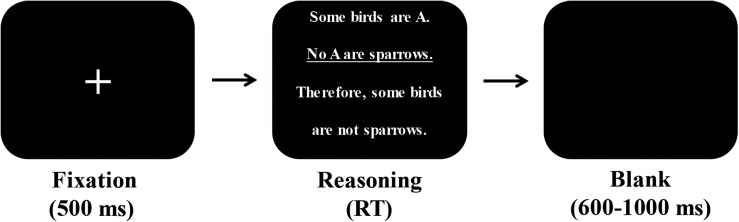
Illustration of the experimental procedure.

The formal experiment consisted of two blocks. The presentation order of these two blocks was counterbalanced across participants. Each trial started with the presentation of a central fixation point with a duration of 500 ms. Subsequently, a categorical syllogism was presented on the screen, and the participants were instructed to press the “F” key with their left index finger if they thought the conclusion logically followed the statements; otherwise, they were asked to press the “J” key with their right index finger. The categorical syllogism remained on the screen until the participants made a response. Following their response, each trial ended with a blank screen that varied randomly from 600 to 1,000 ms (see Figure 2). Within each block, 12 trials were presented in a pseudo-random order; each participant performed 24 trials in total.

Before the formal experiments, participants were asked to complete two practice trials to familiarize themselves with the categorical syllogism. Participants began the formal experiments only after they correctly completed all practice trials. After the formal experiments, they were asked to complete the 18-item NFC scale and the OSPAN task.

## Results

**FIGURE 3:**
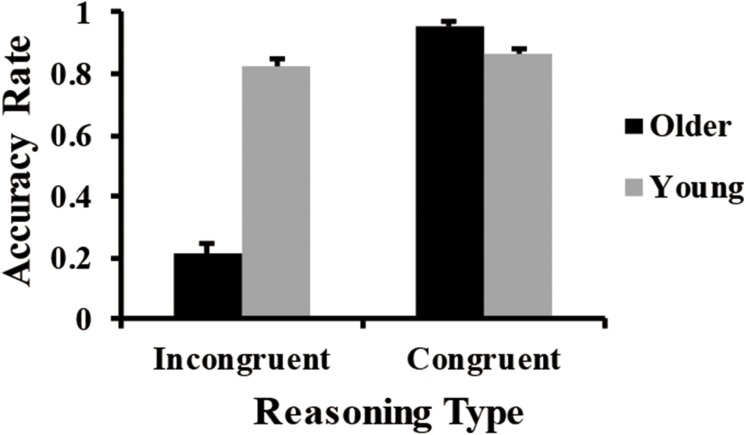
Significant interaction of age group × reasoning type for the accuracy rate. Error bars indicate standard error.

We performed a 2 × 2 repeated-measures analysis of variance (ANOVA) on the accuracy rate (i.e., proportion of times that the participants responded correctly). The main effect of age group was significant, *F*(1,91) = 132.87, *p* < 0.001, $\eta_{P}^{2}$ = .59, and the accuracy rate of young adults (*M* = 0.84, *SD* = 0.10) was significantly higher than that of older adults (*M* = 0.59, *SD* = 0.11). Moreover, the main effect of reasoning type was significant, *F*(1,91) = 345.37, *p* < 0.001, $\eta_{P}^{2}$ = .79, and the accuracy rate in congruent conditions (*M* = 0.91, *SD* = 0.10) was significantly higher than that in incongruent conditions (*M* = 0.53, *SD* = 0.36). Interestingly, a significant interaction effect between age group and reasoning type was observed, *F*(1,91) = 278.47, *p* < 0.001, $\eta_{P}^{2}$ = .75. Follow-up simple effect analyses showed the following. The accuracy rate of older adults (*M* = 0.96, *SD* = 0.14) was significantly higher than that of young adults (*M* = 0.87, *SD* = 0.13) in congruent conditions, *F*(1,91) = 23.31, *p* < 0.001, $\eta_{P}^{2}$ = .20. However, the accuracy rate of older adults (*M* = 0.22, *SD* = 0.28) was significantly lower than that of young adults (*M* = 0.83, *SD* = 0.27) in incongruent conditions, *F*(1,91) = 244.23, *p* < 0.001, $\eta_{P}^{2}$ = .73 (see Figure 3).

In addition, we conducted an analysis with the educational level as a covariate. The results indicated that the accuracy rate of older adults was significantly lower than that of young adults in incongruent conditions, *F*(1,91) = 35.97, *p* < 0.001, $\eta_{P}^{2}$ = .29. The educational level diminished the difference of belief bias between older and young adults in incongruent conditions, *F*(1,91) = 9.77, *p* = 0.002, $\eta_{P}^{2}$ = .10. However, the accuracy rate of older adults was significantly higher than that of young adults in congruent conditions, *F*(1,91) = 7.35, *p* = 0.008, $\eta_{P}^{2}$ = .08. The educational level did not significantly influence the reasoning performance between older and young adults under congruent conditions, *F*(1,91) = 0.04, *p* = 0.841, $\eta_{P}^{2}$ = .00.

We analyzed the logic, belief, and interaction indices for each participant in accordance with standard practice (for a review, see Evans and Curtis-Holmes, 2005). Table 2 shows the mean scores on each index, together with the results of one-sample *t*-tests (one-tailed), which were analyzed to show whether each index was significantly above zero. In older adults, the interaction index was significantly below zero, but the logic index and belief index were non-significant. In young adults, the logic index was significantly above zero, whereas the belief and interaction indices were non-significant.

**TABLE 2:** Computed indices for each condition plus results of one-sample *t* tests (one-tailed).

	Logic index	Belief index	Interaction index
**Older adults (*n* = 45)**			
*Mean*	0.29	–0.33	–8.91
*SD*	1.78	1.85	2.77
*t*(44)	1.09	–1.21	–21.58
*p*	0.289	0.232	<0.001
**Young adults (*n* = 48)**			
*Mean*	1.48	–0.06	–0.48
*SD*	2.56	1.68	2.07
*t*(47)	4.00	–0.26	–1.60
*p*	<0.001	0.798	0.116

We also conducted two-sample *t*-tests to compare the logic and belief indices between older and young adults. The results indicated that the logic index scores of older adults were significantly lower than those of young adults, *t*(91) = −2.59, *p* = 0.011. However, no significant difference was observed in the belief index scores of older and young adults, *t*(91) = 0.74, *p* = 0.461.

### Age Effect Across Problem Types

A 2 (age group: older adults or young adults) × 2 (logic: valid or invalid) × 2 (belief: believable or unbelievable) repeated-measures ANOVA was conducted on the accuracy rate. The results showed that the main effect of age group was significant, *F*(1,91) = 132.87, *p* < 0.001, $\eta_{P}^{2}$ = .59, and the accuracy rate of young adults (*M* = 0.84, *SD* = 0.19) was significantly higher than that of older adults (*M* = 0.59, *SD* = 0.42). Moreover, a significant main effect of logic was observed, *F*(1,91) = 14.77, *p* < 0.001, $\eta_{P}^{2}$ = .14, and the accuracy rate in valid problems (*M* = 0.76, *SD* = 0.35) was significantly higher than that in invalid problems (*M* = 0.68, *SD* = 0.35). However, the main effect of belief was non-significant, *F*(1,91) = 1.17, *p* = 0.280, $\eta_{P}^{2}$ = .01. A two-way interaction between age group and logic was observed, *F*(1,91) = 6.70, *p* = 0.011, $\eta_{P}^{2}$ = .07. Simple effects analyses showed the following. Older adults showed a lower accuracy rate (*M* = 0.60, *SD* = 0.42) than young adults (*M* = 0.91, *SD* = 0.15) in valid problems, *F*(1,91) = 107.01, *p* < 0.001, $\eta_{P}^{2}$ = .54. Similarly, older adults also showed a lower accuracy rate (*M* = 0.57, *SD* = 0.42) than young adults (*M* = 0.78, *SD* = 0.21) in invalid problems, *F*(1,91) = 51.10, *p* < 0.001, $\eta_{P}^{2}$ = .36. The interaction was driven by more pronounced differences between older and young adults in valid problems than in invalid problems. The interaction between age group and belief was non-significant, *F*(1,91) = 0.55, *p* = 0.461, $\eta_{P}^{2}$ = .01. More interestingly, a significant three-way interaction effect was observed, *F*(1,91) = 278.47, *p* < 0.001, $\eta_{P}^{2}$ = .75. Follow-up analyses indicated that no significant difference was observed in the accuracy rate of older adults (*M* = 0.96, *SD* = 0.11) and of young adults (*M* = 0.92, *SD* = 0.13) for valid-believable problems, *F*(1,91) = 1.59, *p* = 0.210, $\eta_{P}^{2}$ = .02. Older adults showed a lower accuracy rate (*M* = 0.24, *SD* = 0.29) than young adults (*M* = 0.89, *SD* = 0.17) for valid-unbelievable problems, *F*(1,91) = 172.06, *p* < 0.001, $\eta_{P}^{2}$ = .65. Moreover, older adults showed a lower accuracy rate (*M* = 0.19, *SD* = 0.21) than young adults (*M* = 0.76, *SD* = 0.24) for invalid-believable problems, *F*(1,91) = 150.62, *p* < 0.001, $\eta_{P}^{2}$ = .62. However, older adults showed a higher accuracy rate (*M* = 0.96, *SD* = 0.10) than young adults (*M* = 0.81, *SD* = 0.18) for invalid-unbelievable problems, *F*(1,91) = 25.33, *p* < 0.001, $\eta_{P}^{2}$ = .22.

### Mediation Analyses

We tested whether NFC and WM mediated the age effect on reasoning performance. We conducted bootstrapping analyses with 1,000 resamples with 95% CIs for the indirect effects. The hypothesized model fitted the data well, CFI = 1.000, TLI = 1.000, RMSEA = 0.000.

**FIGURE 4:**
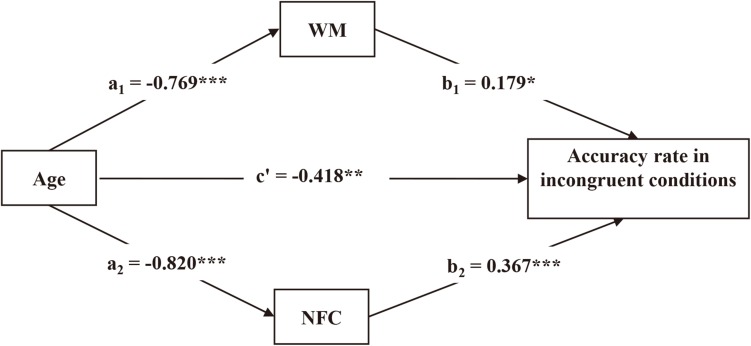
Need for cognition and WM mediated the age effect on reasoning performance under incongruent conditions. ^∗^*p* < 0.05. ^∗∗^*p* < 0.01. ^∗∗∗^*p* < 0.001.

Need for cognition and WM had significant mediating effects in incongruent conditions (β = −0.439, *p* < 0.001), which accounted for 51.2% of the variance. Age was found to have a negative effect on NFC, which in turn had a high significantly positive effect on accuracy rate. Similarly, age was found to have a negative effect on WM, and WM had a positive effect on accuracy rate. The direct age effect on accuracy rate remained significant (see Figure 4).

**FIGURE 5:**
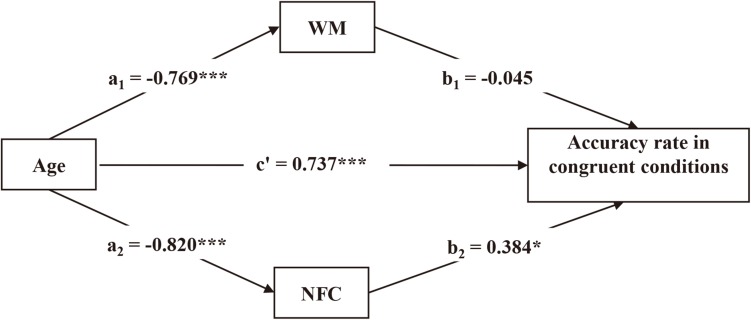
Need for cognition and WM mediated the age effect on reasoning performance under congruent conditions. ^∗^*p* < 0.05, ^∗∗∗^*p* < 0.001.

Need for cognition and WM had marginally significant mediating effects in congruent conditions (β = −0.280, *p* = 0.064). The direction of the direct effect was inverse to the indirect effect, which was attributed to suppressing effects. Therefore, the indirect effect equaled the indirect effect divided by the direct effect rather than the total effect (MacKinnon, 2008; Preacher et al., 2011). The indirect effect accounted for 37.9% of the variance in congruent conditions. Age was found to have a high significantly negative effect on NFC, which in turn had a high significantly positive effect on accuracy rate. Age was found to have a significantly negative effect on WM, and WM had no significant effect on accuracy rate. The direct age effect on accuracy rate was significant (see Figure 5).

## Discussion

The present study showed that older adults had stronger belief bias than young adults in syllogistic reasoning. The accuracy rate of older adults was higher than that of young adults under congruent conditions. Thus, syllogistic reasoning was more significantly boosted by beliefs in older adults compared with young adults under congruent conditions. However, the accuracy rate of older adults was lower than that of young adults under incongruent conditions. Thus, syllogistic reasoning was more significantly hampered by beliefs in older adults compared with young adults under incongruent conditions. Furthermore, WM and NFC mediated the age effect on belief bias under incongruent conditions. However, only NFC mediated the age effect on reasoning performance under congruent conditions. Overall, our findings shed new light on the belief bias effect in syllogistic reasoning.

### Age Effect on Belief Bias

The present study demonstrated that older adults had a stronger belief bias compared with young adults under incongruent conditions, which was consistent with the previous studies. De Neys and Van Gelder (2009) and Tsujii et al. (2010) showed that older adults have a lower accuracy rate than young adults, and they argued that older adults show a more significant belief bias than young adults under incongruent conditions. Moreover, the logic index scores of older adults were significantly lower than those of young adults. On the basis of dual-process theories, older adults are less likely to use analytic strategies and are easily influenced by beliefs. Therefore, older adults undertake syllogistic reasoning depending more on conclusion believability under incongruent conditions, even if conclusion believability conflicts with logical validity. One possible explanation for the age effect in incongruent conditions is that young adults are simply better at processing logical principles and generating the correct responses intuitively than older adults. More precisely, young adults might reason better because they do not need to inhibit rather than because they inhibit better. Some studies have provided evidence for this possible explanation and indicated that good reasoners do not necessarily inhibit erroneous intuitions (Bago and De Neys, 2017, 2019; Newman et al., 2017). Often, they simply have better intuitions and generate the correct response intuitively (De Neys and Pennycook, 2019). Furthermore, this effect seems to be mediated by WM capacity (Thompson et al., 2018), which is known to decline with age.

However, the present study also showed that older adults had a higher accuracy rate compared with young adults under congruent conditions, which differed from the previous studies showing that age does not affect syllogistic reasoning performance under congruent conditions. This inconsistency between the present findings and the previous two studies might be due to the following factors. A key difference is that the average education level of older adults was 14.56 years in the previous studies. However, the average education level of older adults was 9.76 years in the present study. Moreover, Salthouse (2003) indicated that education level might have a modulating effect on cognitive aging. Specifically, the age effect on cognition seemed to be reduced among individuals with a high education level. Consequently, education level might modulate the degree of influence of age on reasoning performance. Another difference is the problem difficulty of syllogistic reasoning. In the present study, the middle item of each syllogism was replaced by the unified English letter “A.” Moreover, the participants were definitely informed that the English letter “A” referred to a nonsensical term, which might lead to simpler syllogistic reasoning for older adults in the present study compared with those in the previous studies. Although the older adults showed a higher accuracy rate than young adults under congruent conditions, it was noteworthy that the age effect was much more pronounced under incongruent conditions.

We found that older adults showed a lower accuracy rate than young adults in valid-unbelievable and invalid-believable problems, whereas older adults showed a higher accuracy rate than young adults in invalid-unbelievable problems. These results are similar to a previous study conducted by Stupple et al. (2011), who found that high-logic and medium-logic groups processed syllogisms more slowly than low-logic groups in valid-unbelievable problems, although there was no difference between high-logic and medium-logic groups. Similarly, the same pattern emerged for the invalid-believable, valid-believable, and invalid-unbelievable problems. Moreover, the group differences were more pronounced in invalid-believable problems compared with the other three problems. Stupple et al. (2011) argued that the relatively long response latencies for invalid-believable problems mainly reflected the performance of normatively responding participants whose diligent analysis of these problems caused the prolonged response latencies. Thus, a plausible explanation for our findings is that older adults were believed to be operating mainly on the basis of a heuristic process and evaluated conclusions according to a rapid and low-effort route. By contrast, young adults were more likely to avoid the analytic processing biases and engage in an assiduous search for counterexample models even when the conclusions were believable and consistent with possible models of the premises.

### Theoretical Contributions and Practical Implications

Our findings extend dual-process theories by showing that belief bias varies between older and young adults. Syllogistic reasoning was more significantly boosted by beliefs in older adults compared with in young adults under congruent conditions. However, syllogistic reasoning was more significantly hampered by beliefs in older adults compared with young adults under incongruent conditions. Moreover, the present study showed that NFC and WM mediated the age effect on reasoning performance. Under incongruent conditions, age was found to have a negative effect on NFC and WM, and they, in turn, had a positive effect on syllogistic reasoning performance. Under congruent conditions, age was found to have a negative effect on NFC and WM, and NFC had a positive effect on syllogistic reasoning performance. However, WM had no effect on syllogistic reasoning performance.

Our findings also have practical implications. Older adults easily believe fake news (Guess et al., 2019). Although this deception is a universal phenomenon in current society, little is known about its potential source. In real life, fake news is often fabricated to appear helpful and beneficial, which leads to older adults suffering from deception. Our findings provide evidence that older adults were easily disturbed by prior knowledge and experiences as cognitive ability and cognitive motivation weakened. In view of this finding, older adults should think positively, apply the principles of logic, and strengthen their cognitive ability and motivation to resist deception when faced with fake news.

### Limitations and Directions for Future Research

Our research has left several open questions for future research. First, although we presented the age effect on belief bias in syllogistic reasoning, we did not fully match education attainment for older and young adults. Further research is needed to investigate the effect of education level on belief bias in older and young adults. Second, our research only examined the belief bias in older and young adults. Future work could attempt to investigate belief bias in syllogistic reasoning across different age groups (e.g., adolescent, middle-aged, and the elderly). Finally, we adopted a classical WM task to measure WM. However, other tasks could also be used to measure WM (e.g., reading span task, see Unsworth et al., 2009). Future research could try to measure WM by using some novel tasks.

## Conclusion

In sum, our findings provide the first evidence that WM and NFC mediate the effect of age on syllogistic reasoning performance. We found that prior beliefs hampered logical reasoning more significantly for older adults than for young adults under incongruent conditions, and boosted logical reasoning more significantly for older adults than for young adults under congruent conditions. We also found that the older the age, the less logical the responses will be, and beliefs affected valid more than invalid syllogisms in older adults. Moreover, we found clear age differences in the accuracy rate for syllogistic reasoning, with invalid-believable problems showing marked variations in accuracy rate relative to other problem types as well as across age groups. These findings revealed that invalid-believable problems are likely to incur the greatest WM demand and require increased NFC relative to other problems.

## Data Availability Statement

All datasets generated for this study are included in the article/Supplementary Material.

## Ethics Statement

The studies involving human participants were reviewed and approved by the Ethics Committee of Hunan Normal University. The patients/participants provided their written informed consent to participate in this study.

## Author Contributions

DD, YC, and XZ designed the research and wrote the manuscript. YC, JL, XC, and MH performed the research. DD, YC, JL, XC, and XZ analyzed the data and were involved in the interpretation of data.

## Conflict of Interest

The authors declare that the research was conducted in the absence of any commercial or financial relationships that could be construed as a potential conflict of interest.

## Supplementary Material

FILE S1 A five-point scale to rate the conclusion believability of the 24 experimental materials; believability ratings on conclusions for the 24 experimental materials.
