# Supplementary material for: Belief Bias Effect in Older Adults: Roles of Working Memory and Need for Cognition
Source: Front Psychol. 2020 Jan 23;10:2940. doi: 10.3389/fpsyg.2019.02940 (PMC6990430; doi:10.3389/fpsyg.2019.02940)
Supplement: FILE S1 — A five-point scale to rate the conclusion believability of the 24 experimental materials; believability ratings on conclusions for the 24 experimental materials. [file Data_Sheet_1.PDF]

5-point scale to rate the conclusion believability of materials

In the pilot study, participants were asked to rate the conclusion believability:

1. How do you think “Some metals are not iron” is believable?

1-very unbelievable 2-unbelievable 3-neutral 4-believable 5-very believable

2. How do you think “Some trees are not willow” is believable?

1-very unbelievable 2-unbelievable 3-neutral 4-believable 5-very believable

3. How do you think “Some animals are not cat” is believable?

1-very unbelievable 2-unbelievable 3-neutral 4-believable 5-very believable

4. How do you think “Some lilies are not flowers” is believable?

1-very unbelievable 2-unbelievable 3-neutral 4-believable 5-very believable

5. How do you think “Some birds are not magpie” is believable?

1-very unbelievable 2-unbelievable 3-neutral 4-believable 5-very believable

6. How do you think “Some milters are not fish” is believable?

1-very unbelievable 2-unbelievable 3-neutral 4-believable 5-very believable

7. How do you think “Some wasps are not insects” is believable?

1-very unbelievable 2-unbelievable 3-neutral 4-believable 5-very believable

8. How do you think “Some flowers are not peony” is believable?

1-very unbelievable 2-unbelievable 3-neutral 4-believable 5-very believable

9. How do you think “Some swallows are not birds” is believable?

1-very unbelievable 2-unbelievable 3-neutral 4-believable 5-very believable

10. How do you think “Some deer are not beasts” is believable?

1-very unbelievable 2-unbelievable 3-neutral 4-believable 5-very believable

11. How do you think “Some animals are not lion” is believable?

1-very unbelievable 2-unbelievable 3-neutral 4-believable 5-very believable

12. How do you think “Some parrots are not birds” is believable?

1-very unbelievable 2-unbelievable 3-neutral 4-believable 5-very believable

13. How do you think “Some birds are not pigeon” is believable?

1-very unbelievable 2-unbelievable 3-neutral 4-believable 5-very believable

14. How do you think “Some crucian carps are not fish” is believable?

1-very unbelievable 2-unbelievable 3-neutral 4-believable 5-very believable

15. How do you think “Some metals are not aluminum” is believable?

1-very unbelievable 2-unbelievable 3-neutral 4-believable 5-very believable

16. How do you think “Some clubs are not flowers” is believable?

1-very unbelievable 2-unbelievable 3-neutral 4-believable 5-very believable

17. How do you think “Some pandas are not animals” is believable?

1-very unbelievable 2-unbelievable 3-neutral 4-believable 5-very believable

18. How do you think “Some flowers are not rose” is believable?

1-very unbelievable 2-unbelievable 3-neutral 4-believable 5-very believable

19. How do you think “Some fish are not carp” is believable?

1-very unbelievable 2-unbelievable 3-neutral 4-believable 5-very believable

20. How do you think “Some flowers are not orchid” is believable?

1-very unbelievable 2-unbelievable 3-neutral 4-believable 5-very believable

21. How do you think “Some butterflies are not worms” is believable?

1-very unbelievable 2-unbelievable 3-neutral 4-believable 5-very believable

22. How do you think “Some fish are not goldfish” is believable?

1-very unbelievable 2-unbelievable 3-neutral 4-believable 5-very believable

23. How do you think “Some banyans are not trees” is believable?

1-very unbelievable 2-unbelievable 3-neutral 4-believable 5-very believable

24. How do you think “Some silver is not metal” is believable?

1-very unbelievable 2-unbelievable 3-neutral 4-believable 5-very believable

Believability ratings on conclusions

Believable	M(SD) young	M(SD) old	t	p	Unbelievable	M(SD) young	M(SD) old	t	p
Some metals are not iron	4.74 (0.74)	4.68 (0.72)	0.39	0.693	Some clubs are not flowers	1.23 (0.91)	1.34 (1.02)	–0.52	0.603
Some trees are not willow	4.87 (0.45)	4.78 (0.53)	0.89	0.378	Some pandas are not animals	1.45 (1.21)	1.61 (1.30)	–0.61	0.545
Some animals are not cat	4.94 (0.32)	4.85 (0.42)	1.04	0.303	Some wasps are not insects	1.36 (0.89)	1.54 (1.00)	–0.87	0.390
Some fish are not goldfish	4.85 (0.63)	4.76 (0.69)	0.67	0.503	Some butterflies are not worms	1.45 (1.18)	1.59 (1.27)	–0.53	0.596
Some birds are not magpie	4.85 (0.66)	4.73 (0.74)	0.79	0.426	Some milters are not fish	1.32 (0.96)	1.46 (0.98)	–0.69	0.487
Some flowers are not orchid	4.79 (0.78)	4.68 (0.85)	0.60	0.550	Some deer are not beasts	1.32 (0.96)	1.51 (1.03)	–0.95	0.347
Some birds are not pigeon	4.96 (0.20)	4.85 (0.36)	1.700	0.093	Some lilies are not flowers	1.62 (1.36)	1.83 (1.38)	–0.73	0.470
Some flowers are not peony	4.77 (0.87)	4.61 (0.97)	0.79	0.427	Some swallows are not birds	1.32 (0.96)	1.49 (1.08)	–0.78	0.439
Some flowers are not rose	4.91 (0.35)	4.78 (0.48)	1.52	0.132	Some parrots are not birds	1.19 (0.71)	1.32 (0.85)	–0.76	0.453
Some animals are not lion	4.96 (0.20)	4.85 (0.36)	1.49	0.138	Some crucian carps are not fish	1.26 (0.89)	1.44 (1.05)	–0.89	0.378
Some metals are not aluminum	4.72 (0.90)	4.56 (1.03)	0.79	0.431	Some silver is not metal	1.47 (1.10)	1.59 (1.10)	–0.50	0.618
Some fish are not carp	4.89 (0.43)	4.71 (0.68)	1.56	0.123	Some banyans are not trees	1.32 (0.98)	1.56 (1.14)	–1.07	0.288
